# Supplementary material for: Escherichia coli adhesion portion FimH functions as an adjuvant for cancer immunotherapy
Source: Nat Commun. 2020 Mar 4;11:1187. doi: 10.1038/s41467-020-15030-4 (PMC7055316; doi:10.1038/s41467-020-15030-4)
Supplement: Supplementary file 2 — Supplementary Information [file 41467_2020_15030_MOESM2_ESM.pdf]

Supporting Information for

Escherichia coli adhesion portion FimH functions as an adjuvant for cancer immunotherapy

Wei et al,.

Fig. S1

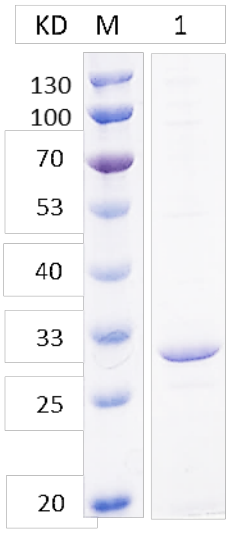

Supplementary figure 1. Purification of recombinant FimH from *E. coli*. pET28a-FimH plasmids were transfected into competent cells and the FimH protein was purified on a Nickel column. Lane 1 shows the purified FimH separated on SDS-PAGE; M = molecular weight marker.

Fig. S2

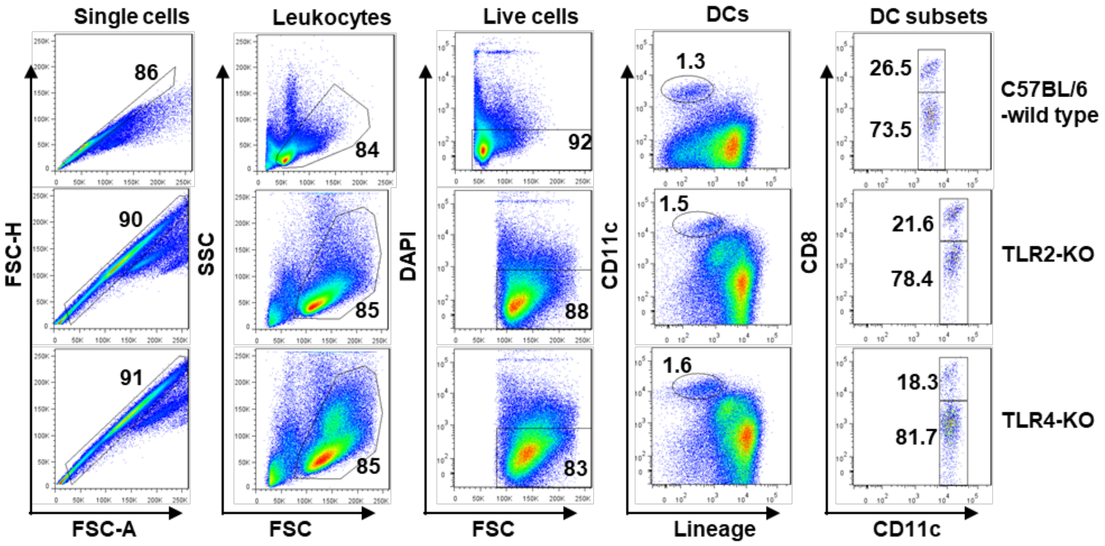

Supplementary figure 2. Definition of dendritic cells (DCs) in inguinal lymph node (iLN). iLN was harvested from C57BL/6, TLR2-KO and TLR4-KO mice and the cells were

stained with a lineage marker and CD11c. The lineage markers included were anti-B220, anti-CD3, anti-CD49b, anti-Gr1, anti-Thy1.1, and anti-TER-119. $Lineage^{-}CD11c^{+}$ live leukocytes were defined as DCs. The DCs were further divided into $CD8\alpha^{+}$ and $CD8\alpha^{-}$ DCs.

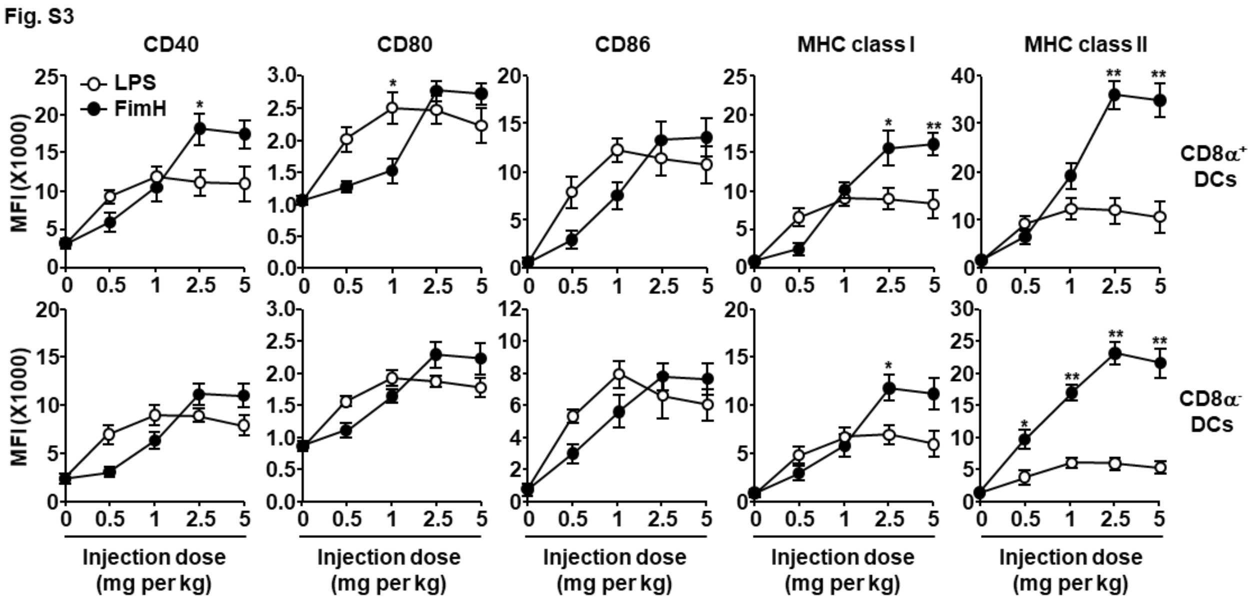

Supplementary figure 3. Dose-dependent effect of FimH in mouse DC activation. C57BL/6mice were injected subcutaneously (*s.c.*) with the indicated dose of FimH or LPS for 24 h and the iLN was harvested. The expression level of costimulatory molecules and MHC classes I and II in $CD8\alpha^{+}$ DCs (upper panel) and $CD8\alpha^{-}$ DCs (lower panel) are shown. n = 4. All data are presented as mean ± SEM. $*p < 0.05$, $**p < 0.01$.

Fig. S4

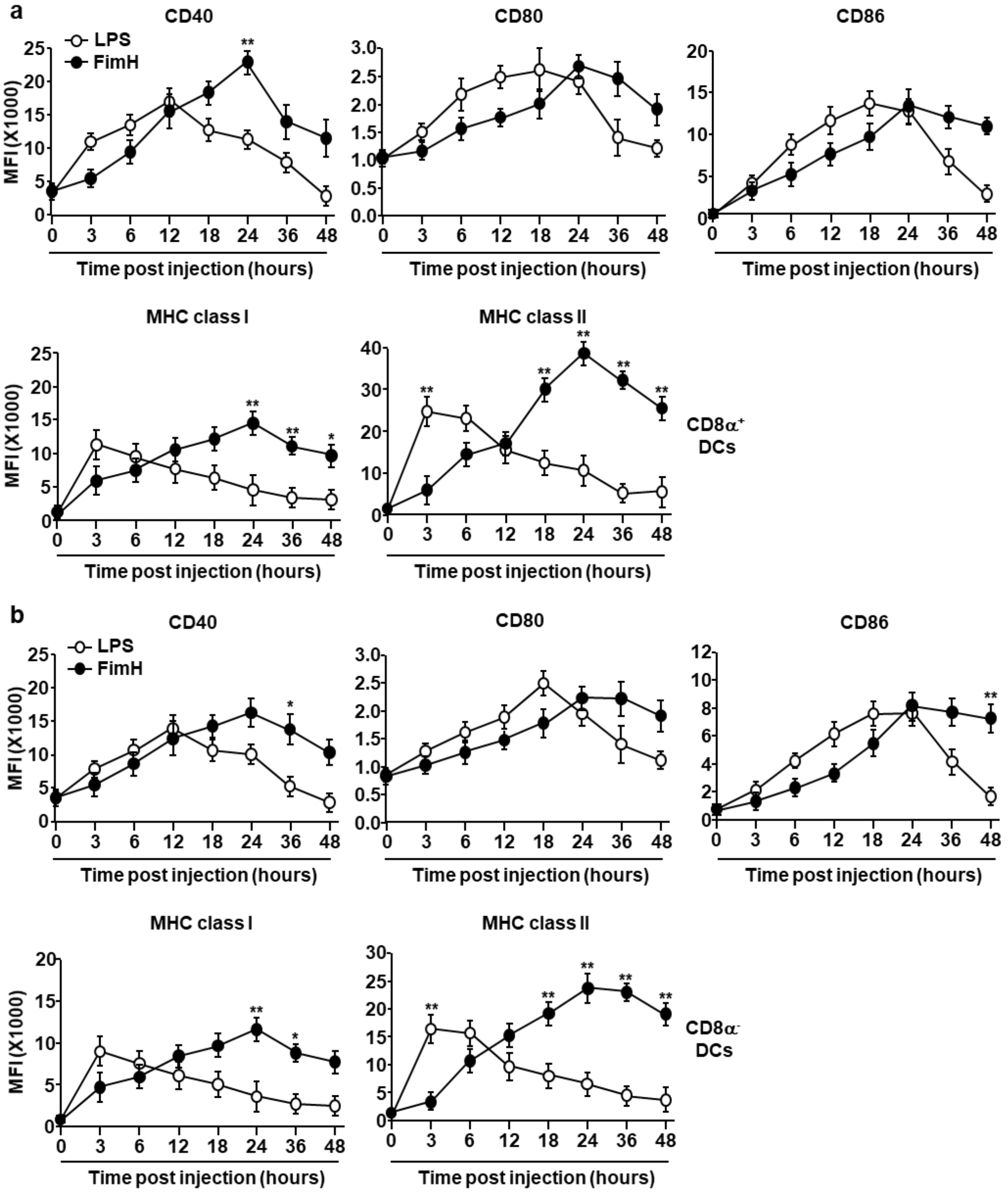

Supplementary figure 4. Time-dependent effect of FimH on the activation of mou se iLN DCs. C57BL/6 mice were injected *s.c.* with 2.5 mg per kg of FimH and 1 mg per kg of LPS and iLN was harvested as indicated by the time points post-injection. The expression level of costimulatory molecules and MHC classes I and II in (**a**) CD8α$^+$ DCs and (**b**) CD8α$^-$ DCs are shown. n = 4. All data are presented as mean ± SEM. *$p < 0.05$, **$p < 0.01$.

Fig. S5

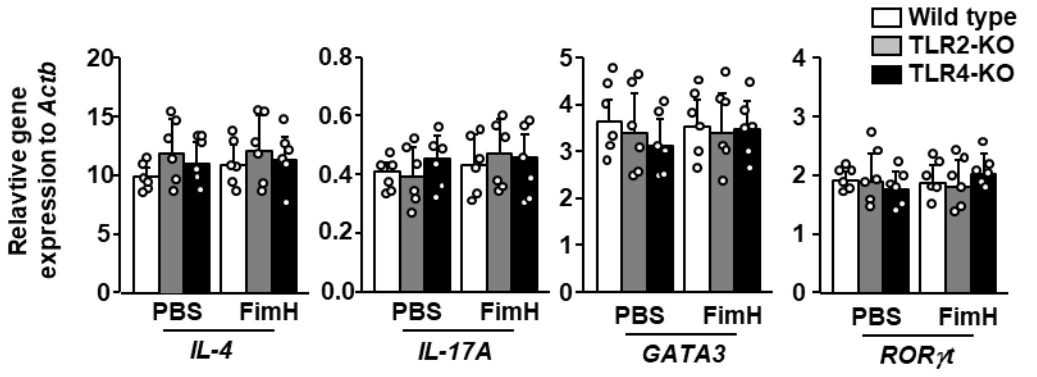

Supplementary figure 5. FimH does not promote Th2 and Th17 immune responses. C57BL/6, TLR2-KO, and TLR4-KO mice were injected with 2.5 mg per kg FimH. Twenty-four hours after treatment, the iLN was harvested and analyzed to estimate mRNA levels. n = 6. All data are presented as mean ± SEM.

Fig. S6

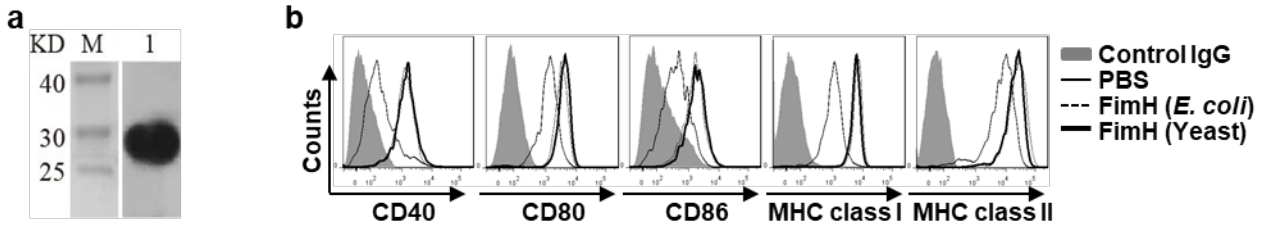

Supplementary figure 6. FimH from yeast had induced activation of DCs. FimH was purified from yeast, as indicated in the “Materials and Methods” section. **a** Lane 1 demonstrates the recombinant FimH extracted from yeast, M = molecular weight marker. **b** The FimH from *E. coli* or yeast were injected to C57BL/6 mice and DC activation was analyzed in iLN. The expression of costimulatory molecules and MHC class I and II are shown. n = 6. All data are presented as mean ± SEM.

Fig. S7

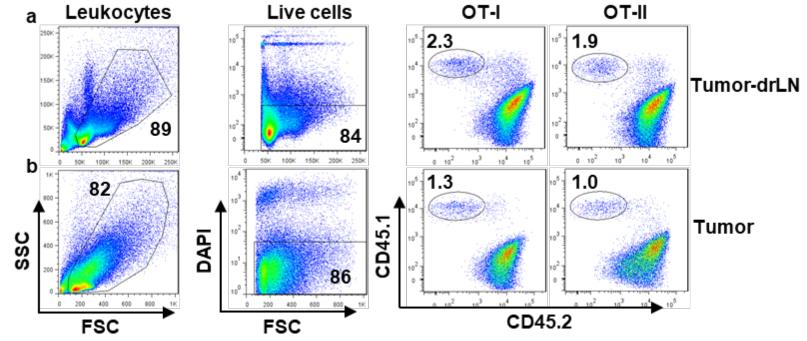

Supplementary figure 7. Flow cytometry analysis of OT-I and OT-II cells in tumor-draining lymph node and tumor. Analysis strategy of flow cytometry for detection of CD45.1-expressing OT-I and OT-II cells in CD45.2 congenic mice (a) tumor-drLN and (b) tumor was shown.

Fig. S8

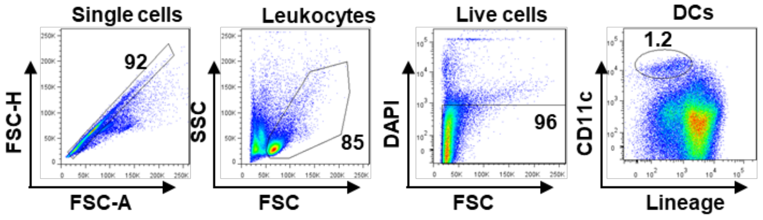

Supplementary figure 8. Definition of DCs in mediastinal lymph node (mLN). mLN was harvested from C57BL/6 and the cells were stained as shown in supplemental figure 2. Lineage$^-$CD11c$^+$ live leukocytes were defined as DCs in the mLN.

Fig. S9

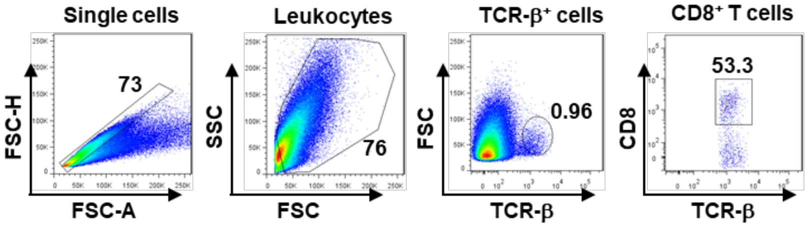

Supplementary figure 9. Gating strategy for tumor-infiltrated CTLs by combination treatment of FimH and anti-PD-L1 antibodies. CTLs in CT26 tumors were analyzed by

flow cytometry. Strategy of flow cytometry analysis for CTLs in CT26 tumor was shown.

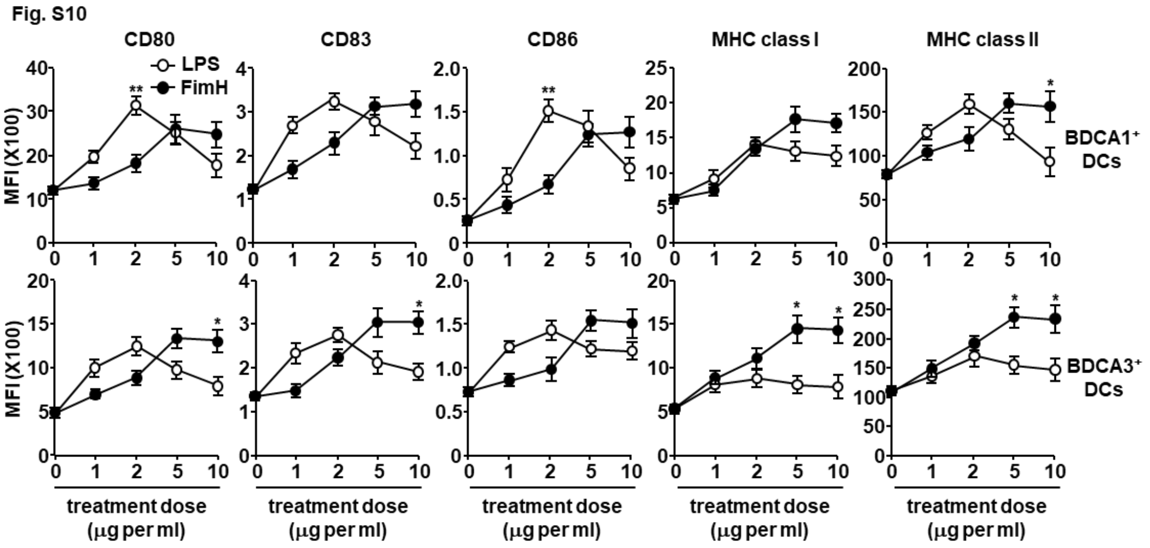

Supplementary figure 10. Dose-dependent effect of FimH in the activation of human peripheral blood DCs (PBDCs) in a dose-dependent manner. Peripheral blood mononuclear cells (PBMCs) were treated with the indicated dose of FimH or LPS for 24 h. The expression levels of costimulatory molecules and MHC classes I and II in $BDCA1^{+}$ (upper panel) and $BDCA3^{+}$ DCs (lower panel) are measured by flow cytometry. n = 4. All data are presented as mean ± SEM. *$*p < 0.05$, $**p < 0.01$.*

Fig. S11

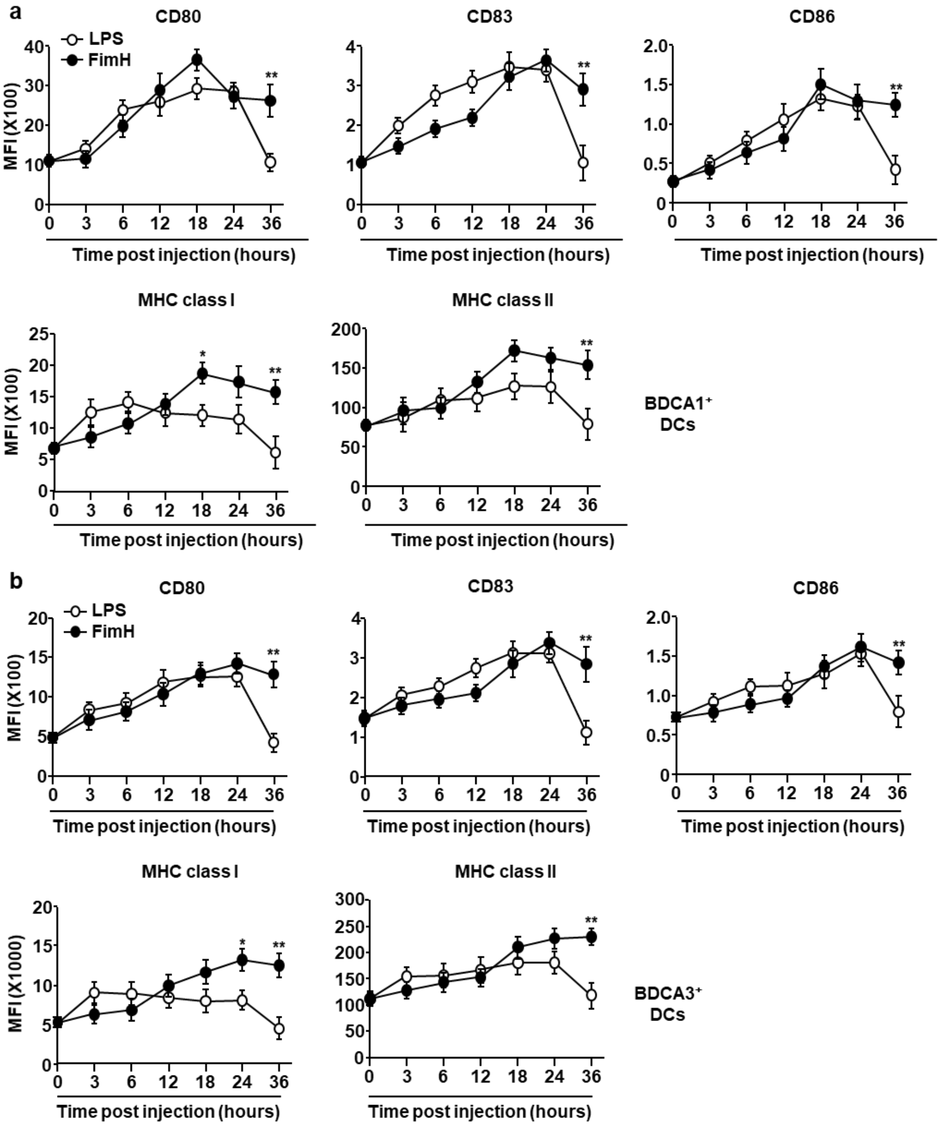

Supplemental figure 11. Time-dependent effect of FimH in the activation of huma n PBDC. PBMCs were treated with 5 μg per ml of FimH and 2 μg per ml of LPS, and were harvested as indicated by the time point. The expression levels of costimulatory molecules and MHC classes I and II in (**a**) BDCA1$^+$ DCs n = 4 and (**b**) BDCA3$^+$ DCs n = 4 are shown. All data are presented as mean ± SEM. *$p < 0.05$, **$p < 0.01$.*
